# No Difference in Liver Damage Induced by Isocaloric Fructose or Glucose in Mice with a High-Fat Diet

**DOI:** 10.3390/nu16203571

**Published:** 2024-10-21

**Authors:** Wei-Fan Hsu, Ming-Hsien Lee, Chong-Kuei Lii, Cheng-Yuan Peng

**Affiliations:** 1 Center for Digestive Medicine, Department of Internal Medicine, China Medical University Hospital, Taichung 404327, Taiwan; 2 Graduate Institute of Biomedical Sciences, China Medical University, Taichung 404333, Taiwan; 3 School of Chinese Medicine, China Medical University, Taichung 404328, Taiwan; 4 Metabolic and Bariatric Surgical Department, Taichung Tzu Chi Hospital, Taichung 427003, Taiwan; 5 Department of Nutrition, China Medical University, Taichung 404328, Taiwan; 6 School of Medicine, China Medical University, Taichung 406040, Taiwan

**Keywords:** ceramides, fructose, glucose, high-fat diet, metabolic dysfunction-associated steatotic liver disease, metabolic dysfunction-associated steatohepatitis

## Abstract

**Background/Objectives**: The diverse effects of fructose and glucose on the progression of metabolic dysfunction-associated steatotic liver disease remain uncertain. This study investigated the effects, in animal models, of high-fat diets (HFDs) supplemented with either glucose or fructose. **Methods**: Six-week-old, male C57BL/6J mice were randomly allocated to four groups: normal diet (ND), HFD, HFD supplemented with fructose (30% *w*/*v*, HFD + Fru), and HFD supplemented with glucose (initially 30%, HFD + Glu). After 24 weeks, liver and plasma samples were gathered for analysis. In addition, 39 patients with obesity undergoing bariatric surgery with wedge liver biopsy were enrolled in the clinical study. **Results**: The HFD + Glu group consumed more water than did the HFD and HFD + Fru groups. Thus, we reduced the glucose concentration from 30% at baseline to 15% at week 2 and 10% starting from week 6. The HFD + Fru and HFD + Glu groups had a similar average caloric intake (*p* = 0.463). The HFD increased hepatic steatosis, plasma lipid levels, lipogenic enzymes, steatosis-related oxidative stress, hepatic inflammation, and early-stage liver fibrosis. Supplementation with fructose or glucose exacerbated liver damage, but no significant differences were identified between the two. The expression patterns of hepatic ceramides in HFD-fed mice (with or without supplemental fructose or glucose) were similar to those observed in patients with obesity and severe hepatic steatosis or metabolic dysfunction–associated steatohepatitis. **Conclusions**: Fructose and glucose similarly exacerbated liver damage when added to an HFD. Ceramides may be involved in the progression of hepatic lipotoxicity.

## 1. Introduction

The prevalence of nonalcoholic fatty liver disease (NAFLD) is escalating, and the condition has become a significant public health issue. Nonalcoholic steatohepatitis (NASH), the aggressive form of NAFLD, has evolved to be the fastest-growing reason for liver transplantation in both America [1] and Europe [2]. Histologically, NAFLD is characterized by the storing of triglyceride fat droplets > 5% of the liver parenchyma [3]. NAFLD manifests a series of hepatic injuries, progressing from simple steatosis to NASH, which involves hepatic necroinflammation and fibrosis, and potentially advancing to liver cirrhosis and hepatocellular carcinoma [4]. The term metabolic dysfunction-associated steatotic liver disease (MASLD) was introduced to better describe the clinical manifestations of cardiometabolic criteria in patients with fatty liver disease in 2023 [5]. Furthermore, metabolic dysfunction-associated steatohepatitis (MASH) has been proposed as a replacement for NASH.

Hepatic steatosis and lipotoxicity are key contributors to the progression of MASLD [6]. The severity of liver disease in patients with MASH is connected to elevated levels of saturated fatty acids [7]. Additional factors that can induce hepatic inflammation and fibrosis include inflammatory cytokines, mitochondrial dysfunction, adipokines, endoplasmic reticulum stress, and oxidative stress [8]. The “multiple-hit pathogenesis” has now replaced the “two-hit hypothesis” model [6]. Sugar-sweetened beverages, which constitute the most significant origin of added sweetening in food (accounting for 15% of total energy intake), substantially lead to the occurrence and progression of MASLD [9]. Glucotoxicity and lipotoxicity are critical factors in the progression of MASLD [6]. Carbohydrates play a crucial role in this process as the primary infrastructure for hepatic de novo lipogenesis (DNL) [10]. Excessive consumption of free sugars is highly associated with the incidence and worsening of MASLD [9,11,12]. However, whether different types of sugars, such as glucose and fructose, have diverse effects on MASLD progression remains unclear. Human interventional studies comparing the effects of fructose and glucose on hepatic fat accumulation and insulin resistance have not reported significant differences [13,14,15]. Thus, the debate over the distinct effects of glucose and fructose on MASLD deterioration continues.

Ceramides, which are components of sphingolipids, are lipotoxic lipids. Sphingolipids are essential constituents of cell membranes and function as potent signaling molecules that regulate diverse cellular processes. Hydrolysis of sphingomyelins is the primary source of ceramide, and ceramide can also come from the re-acylation of sphingosine (salvage pathway) and the synthesis of serine and palmitoyl-CoA (de novo synthesis) [16]. Increased levels of total hepatic ceramides, including C16:0, C22:0, and C24:1 dihydroceramides, were observed in patients with MASH [17]. These ceramides are key mediators of saturated fatty acid lipotoxicity [18]. In addition, diet-induced MASLD mice fed a Western diet combined with sugar water exhibited hepatic steatosis along with increased levels of hepatic ceramides [19].

The aim of this study is to investigate the differential effects of fructose and glucose on the progression of MASLD in animal models fed a high-fat diet (HFD) supplemented with either glucose or fructose. In addition, this study examined the expression of ceramides in both these animal models and in humans with obesity.

## 2. Materials and Methods

### 2.1. Animal Protocol

We purchased 5-week-old male C57BL/6J mice from the National Laboratory Animal Center (Taipei, Taiwan) and bred the mice in the Animal Center of China Medical University. The mice were randomly allocated to four groups (*n* = 12 per group) after a 1-week acclimation period. The mice were retained at 22 °C and had free access to food and water in a 12-h light–dark cycle during the acclimation. The mice were separated into four groups: normal diet [ND] and three HFD subgroups after acclimation. The ND group was fed a chow diet (D12450J, Research Diets, New Brunswick, NJ, USA), and the HFD group was fed an HFD (D12492, Research Diets) for 24 weeks. The chow diet consisted of 20% protein, 10% fat, and 70% carbohydrates (125 g Lodex 10 and 72.8 g sucrose in total 773.85 g chow diet) and had an energy density of 3.82 kcal/g; the HFD consisted of 20% protein, 27% fat (24.5% lard and 2.5% soybean oil), and 20% carbohydrates, and had an energy density of 5.21 kcal/g (60% energy from fat). The mice in the HFD group received either tap water (HFD group), 30% (*w*/*v*) fructose in water (HFD + Fru group), or 30% glucose in water (HFD + Glu group) [20]. The mice were weighed weekly. Metabolic parameters were measured at the Institutional Animal Care Facility of China Medical University in vivo. The Institutional Animal Care and Use Committee of China Medical University approved this animal study (CMUIACUC-2020-071) on 30 December 2019, following the *Guide for the Care and Use of Laboratory Animals* published by the National Academy of Sciences (USA).

The animals were fasted overnight after 24 weeks of feeding, and they were anesthetized with isoflurane and then sacrificed by guillotine. Blood was obtained from the tail vein before sacrifice. Plasma samples were gathered from the blood supernatant centrifuged at 3000× *g* for 10 min at 4 °C and reserved at −80 °C until analysis. The liver was rapidly removed, the weight was measured following ice-cold phosphate-buffered saline perfusion, and then aliquoted. Portions of the liver were snap-frozen in liquid nitrogen and saved at −80 °C for further investigation. A section of the liver was also embedded in 10% formalin for pathological examination.

### 2.2. Biochemical Analysis

Alanine aminotransferase (ALT), triglycerides, total cholesterol, high-density lipoprotein cholesterol (HDL-C), and low-density lipoprotein cholesterol (LDL-C) in the plasma were measured by Diasys kits (Holzheim, Germany). Glucose and insulin levels were measured by Randox (Kearneysville, WV, USA) and Mercodia kits (Uppsala, Sweden), respectively, in accordance with the producer’s instructions. Homeostasis model assessment of insulin resistance (HOMA-IR) was performed by following the formula to measure insulin resistance [21]:

HOMA-IR = insulin (μU/mL) × glucose (mg/dL)/405

### 2.3. Histological Analyses

Liver samples were fixed overnight in 4% formaldehyde and then implanted in paraffin for sectioning. The staining of hematoxylin and eosin was performed on the liver sections to evaluate general tissue morphology. Masson’s trichrome staining was performed to assess the degree of hepatic fibrosis. A light microscope (Leica DMi8, Wetzlar, Germany) gathered histological images of tissue sections. For quantitative analysis, seven fields of interest were randomly selected per slide, and the average values from these fields were calculated for each slide. The severity of liver steatosis, presence of MASH, and severity of hepatic fibrosis were evaluated in accordance with the criteria established by the NASH Clinical Research Network (CRN) [22].

### 2.4. Real-Time Quantitative Polymerase Chain Reaction

Reverse transcription of 1 μg of total RNA isolated using TRIzol (Invitrogen, Carlsbad, CA, USA) synthesized complementary DNA (cDNA), which was purified by the Aurum Total RNA Fatty and Fibrous Tissue Kit (Bio-Rad, Hercules, CA, USA). Table S1 lists the primer sequences used in real-time quantitative polymerase chain reactions (qPCRs). Quantitative gene expression was examined using a real-time thermocycler (MiniOpticon, Bio-Rad, Hercules, CA, USA). The calculation of 2^−ΔΔCt^ formula was performed to identify each mRNA’s relative expression levels, and glyceraldehyde-3-phosphate dehydrogenase mRNA served as the reference [23].

### 2.5. Western Blot in Animal and In Vitro Studies

Tissues were lysed, and protein extracts were prepared as described elsewhere [24]. Equal amounts of protein from the liver homogenates of each group were separated through electrophoresis by using sodium dodecyl sulfate–polyacrylamide gels and subsequently transferred onto polyvinylidene difluoride membranes (Millipore, Billerica, MA, USA). The 5% nonfat dry milk in Tris (15 mM)/NaCl (150 mM) buffer (pH 7.4) was used to block the membranes at room temperature for 2 h. After blocking, the membranes were immersed with appropriate primary antibodies, followed by horseradish peroxidase-conjugated secondary antibodies. An enhanced chemiluminescence kit (Bio Kit, Miaoli, Taiwan) was used, and a luminescence image analyzer (Fuji Film LAS-4000, Tokyo, Japan) scanned and visualized the protein bands [25]. The intensities of the bands of interest were measured using Image J (1.50d, National Institute of Mental Health, Bethesda, MD, USA).

### 2.6. Determination of Lipid Peroxidation in the Liver

Liver homogenates (prepared as 10% *w*/*v* of liver tissue in 1.15% KCl) were centrifuged at 10,000× *g* for 15 min at 4 °C. Measurement of lipid peroxide levels was detected by thiobarbituric acid-reactive substance (TBARS) values in the resulting supernatant following the method described previously [26].

### 2.7. Analysis of Sphingolipids

Liver specimens were frozen at −80 °C until use. The samples were homogenized by grinding in a mortar with liquid nitrogen before extraction. For lipid extraction, we followed the procedure reported by Bligh and Dyer [27] with some modifications. Sphingolipids were analyzed using an Agilent 1290 ultra-high-performance liquid chromatography system coupled with an Agilent 6460 triple quadrupole liquid chromatograph–mass spectrometer (LC–MS; Agilent Technologies, Santa Clara, CA, USA). Separations were performed on an Agilent Eclipse Plus C18 column (100 × 2.1 mm, 1.8 μm; Agilent Technologies). The mobile phase consisted of solvent A (water, methanol, and formic acid in a 40:60:0.2 ratio [*v*/*v*] with 10 mM ammonium acetate) and solvent B (LC–MS-grade isopropanol, methanol, and formic acid in a 40:60:0.2 ratio [*v*/*v*] with 10 mM ammonium acetate). The flow rate was set at 0.35 mL/min. The gradient elution program was performed in the following way: 0–2 min linear gradient from 35% to 80% solvent B; 2–7 min linear gradient from 80% to 100% solvent B, and hold at 100% solvent B for 7 min. Sphingolipids were analyzed using electrospray ionization in positive ion mode with the following parameters: dry gas flow rate, 7 L/min; dry gas temperature, 325 °C; sheath gas temperature, 325 °C; nebulizer pressure, 35 psi; capillary voltage, 3500 V; sheath gas flow rate, 11 L/min; and nozzle voltage, 500 V. Sphingolipids were measured in the precursor ion scan mode by scanning the product ions of *m*/*z* 636.6 to 264.3 for ceramides (longer carbon chain with higher initial *m*/*z*). Multiple reaction monitoring modes were used to confirm sphingolipids and other single-chain sphingolipids further. An internal standard solution containing 100 ng/mL C18 ceramide-d7 (d18:1-d7/18:0) was prepared in methanol/chloroform (2:1, *v*/*v*) [28,29].

### 2.8. Chemicals

Fetal bovine serum was obtained from Gibco Laboratory (Grand Island, NY, USA). The following antibodies were applied in the study: sterol regulatory element-binding protein (SREBP)-1c (NB600−582) from Novus (Littleton, CO, USA); acetyl-CoA carboxylase (ACC, #3676), fatty acid synthase (FAS, #3180), and glutamate–cysteine ligase catalytic subunit (GCLC, ab41463) from Abcam (Cambridge, MA, USA); glutamate–cysteine ligase modifier subunit (GCLM, sc22754) and C/EBP homologous protein (CHOP, sc-61) from Santa Cruz (Santa Cruz, CA, USA); phosphorylated nuclear factor-erythroid 2 (pNrf2, PA5-67520) from Invitrogen (Carlsbad, CA, USA); peroxisome proliferator-activated receptor (PPAR-α, #2443) from Cell Signaling (Danvers, MA, USA); superoxide dismutase 2 (SOD-2, #06-984) and heme oxygenase 1 (HO-1, #374090) from Merck Millipore (Billerica, MA, USA); glutathione peroxidase 2 (GPX2, GTX100292), catalase (GTX110704), α-smooth muscle actin (α-SMA, GTX1000034), and glyceraldehyde-3-phosphate dehydrogenase (GAPDH, #MAB374) from Gene Tex (San Antonio, TX, USA); and nucleotide-binding oligomerization domain leucine-rich repeat and pyrin domain containing 3 (NLRP3, #15101) from Cell Signaling (Danvers, MA, USA).

### 2.9. Clinical Study

Bariatric surgery results in the amelioration of steatosis, MASH, and liver fibrosis [30,31]. In this study, thirty-nine consecutive patients with morbid obesity who had either a body mass index (BMI) of ≥40 kg/m^2^ or a BMI of ≥35 kg/m^2^ plus a metabolic disorder were enrolled. During bariatric surgery, a wedge liver biopsy was performed on the edge of the right lobe. Baseline data were collected, namely BMI, comorbidities, and hematologic and biochemical parameters. The severity of hepatic steatosis, presence of MASH, and liver fibrosis were assessed in accordance with the criteria established by the NASH Clinical Research Network [22]. Hematologic analyses were conducted using a Sysmex HST series system (Kanagawa, Japan), and biochemical analyses were performed using Beckman Coulter instruments (Brea, CA, USA) in the central laboratory of China Medical University Hospital.

The Research Ethics Committee of China Medical University Hospital, Taichung, Taiwan, approved the clinical study (CMUH108-REC1-169) on 20 February 2020, in accordance with ethical guidelines and the principles outlined in the 1975 Declaration of Helsinki. All patients provided written informed consent before enrolment.

### 2.10. Statistical Analyses

In the clinical study, the median (first–third quartile) is used to express continuous variables, and a Mann–Whitney U test was used to compare between groups. In the rodent study, mean ± standard deviation was used to present data. Data obtained from the Agilent triple quadrupole LC–MS were saved in comma-separated values files and processed using Microsoft Excel (version 2409). Integrated peak areas were normalized by sample wet weights to account for potential differences in sample size. Statistical significance in each group was determined using one-way analyses of variance, followed by Bonferroni post hoc tests. All statistical analyses were conducted using SPSS (version 25.0, IBM, Armonk, NY, USA). A two-sided *p*-value of <0.05 was considered statistically significant.

## 3. Results

### 3.1. Effects of HFD and HFD Supplemented with Fructose or Glucose on Body Weight Gain in Mice

**Figure 1:**
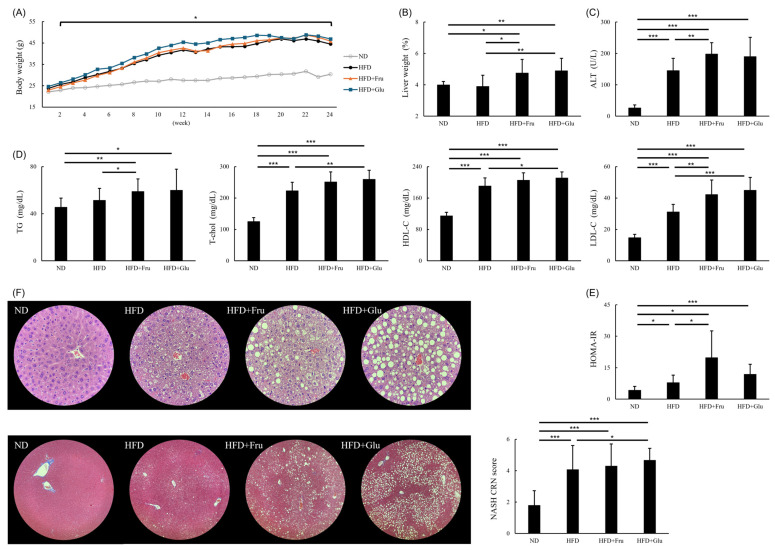
Effects of HFD and HFD supplemented with fructose or glucose on body and liver weight, biochemical parameters, and liver histology. (**A**) Body weight (* *p* < 0.05 compared with ND group). (**B**) Liver weight as a percentage of body weight. (**C**) ALT levels. (**D**) TG, T-chol, HDL-C, and LDL-C levels. (**E**) Insulin resistance. (**F**) Liver histology: hematoxylin and eosin staining (400×, upper panel), Masson’s trichrome staining (200×, lower panel), and NASH CRN score. ND, normal diet; HFD, high-fat diet; HFD + Fru, high-fat diet supplemented with fructose; HFD + Glu, high-fat diet supplemented with glucose; ALT, alanine aminotransferase; HDL-C, high-density lipoprotein cholesterol; HOMA-IR, homeostasis model assessment-insulin resistance; LDL-C, low-density lipoprotein cholesterol; NASH CRN, nonalcoholic steatohepatitis Clinical Research Network; T-chol, total cholesterol; TG, triglyceride. * *p* < 0.05, ** *p* < 0.01, *** *p* < 0.001.

The mean daily dietary intake per mouse during the study duration was 2.33 g in the ND group, 2.13 g in the HFD group, 1.87 g in the HFD + Fru group, and 1.83 g in the HFD + Glu group (Figure S1A). The mean daily drinking water was 2.64 g in the ND group, 2.41 g in the HFD group, 2.47 g in the HFD + Fru group, and 7.49 g in the HFD + Glu group (Figure S1B). Because the HFD + Glu group consumed substantially more water and thus more calories than did the HFD and HFD + Fru groups, we reduced the glucose concentration from 30% at baseline to 15% at week 2 and then to 10% starting from week 6. The average daily calorie intake levels were 8.84 ± 1.15, 11.06 ± 1.29, 12.68 ± 1.45, and 12.94 ± 1.71 kcal/day in the ND, HFD, HFD + Fru, and HFD + Glu groups, respectively (Figure S1C). The HFD group had a significantly higher total calorie intake than did the ND group (*p* < 0.001), and the HFD + Fru and HFD + Glu groups had a significantly higher total calorie intake than did the HFD group (*p* < 0.001). No significant difference in average calorie intake was observed between the HFD + Fru and HFD + Glu groups (*p* = 0.463). Table S2 lists the amounts of food and water consumed per mouse in each group. After 2 weeks of feeding, the body weights in the HFD, HFD + Fru, and HFD + Glu groups were significantly heavier than those in the ND group, and this difference persisted throughout the 24-week period. No significant differences in body weights were noted among the HFD, HFD + Fru, and HFD + Glu groups (Figure 1A).

The percentage of liver weight relative to body weight was similar between the HFD and ND groups and higher in the HFD + Fru and HFD + Glu groups than in the ND and HFD groups (Figure 1B).

### 3.2. Effects of HFD and HFD Supplemented with Fructose or Glucose on Blood Biochemical Parameters

After 24 weeks of feeding, the HFD group had higher levels of ALT (Figure 1C), total cholesterol, HDL-C, LDL-C (Figure 1D), and greater insulin resistance (Figure 1E) than did the ND group. The HFD + Fru and HFD + Glu groups had higher levels of ALT (Figure 1C), triglycerides, total cholesterol, HDL-C, and LDL-C (Figure 1D) and greater insulin resistance (Figure 1E) than did the HFD group. No differences in biochemical parameters were observed between the HFD + Fru and HFD + Glu groups.

### 3.3. Effects of HFD and HFD Supplemented with Fructose or Glucose on Histology

Histological examination revealed steatosis in more than 5% of hepatocytes in the HFD, HFD + Fru, and HFD + Glu groups. In addition, hepatocellular ballooning was observed in the HFD, HFD + Fru, and HFD + Glu groups. However, lobular inflammation was minimal, and no substantial hepatic fibrosis was detected (Figure 1F). Table S3 lists the NASH CRN scores for each group.

### 3.4. Effects of HFD and HFD Supplemented with Fructose or Glucose on Lipogenic Enzymes, Oxidative Stress, and Lipid Peroxidation

**Figure 2:**
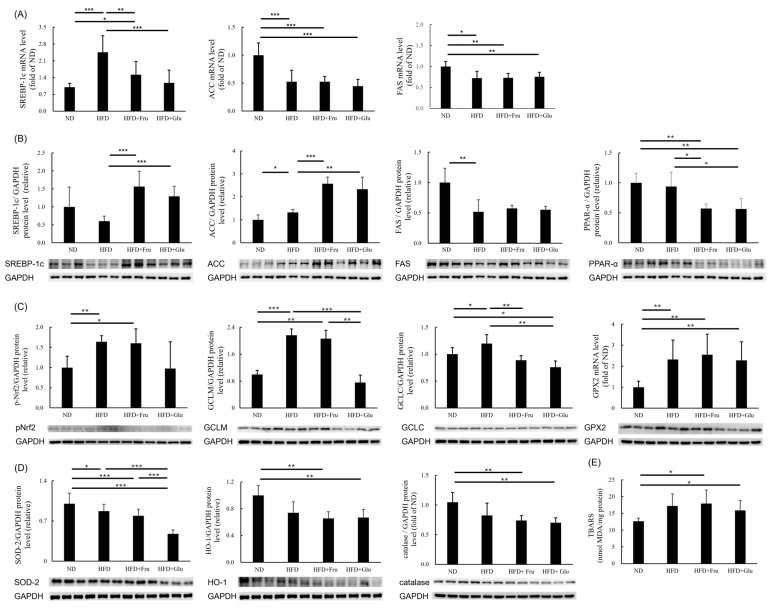
Effects of HFD and HFD supplemented with fructose or glucose on lipogenic enzymes, oxidative stress markers, and lipid peroxidation. (**A**) mRNA levels of SREBP-1c, ACC, and FAS. (**B**) Protein levels of SREBP-1c, ACC, FAS, and PPAR-α. (**C**) Protein levels of p-Nrf2, GCLM, GCLC, and GPX2. (**D**) Protein levels of SOD-2, HO-1, and catalase. (**E**) TBARS values. ND, normal diet; HFD, high-fat diet; HFD + Fru, high-fat diet supplemented with fructose; HFD + Glu, high-fat diet supplemented with glucose; ACC, acetyl-CoA carboxylase; FAS, fatty acid synthase; GCLC, glutamate–cysteine ligase catalytic subunit; GCLM, glutamate–cysteine ligase modifier subunit; GPX2, glutathione peroxidase 2; HO-1, heme oxygenase 1; pNrf2, phosphorylated nuclear factor-erythroid 2; PPAR-α, peroxisome proliferator-activated receptor α; SOD-2, superoxide dismutase 2; SREBP-1c, sterol regulatory element-binding protein-1c; TBARS, thiobarbituric acid-reactive substance. * *p* < 0.05, ** *p* < 0.01, *** *p* < 0.001.

The HFD group displayed higher SREBP-1c mRNA expression and decreased ACC and FAS mRNA expression compared with the ND group. Furthermore, the HFD + Fru and HFD + Glu groups exhibited decreased ACC and FAS mRNA expressions compared with the ND group (Figure 2A). The HFD group displayed higher ACC protein levels compared with the ND group, and the HFD + Fru and HFD + Glu groups exhibited increased protein levels of SREBP-1c and ACC compared with the HFD group. The HFD group exhibited decreased FAS protein levels compared with the ND group (Figure 2B). The HFD + Fru and HFD + Glu groups displayed decreased PPAR-α protein levels compared with the ND and HFD groups (Figure 2B, right panel).

The HFD and HFD + Fru groups exhibited increased protein levels of p-Nrf2, GCLM, and GCLC compared with the ND group. However, the HFD + Glu group did not exhibit higher levels of these proteins compared with the ND group (Figure 2C). Furthermore, the HFD, HFD + Fru, and HFD + Glu groups displayed increased protein levels of GPX2 compared with the ND group (Figure 2C, right panel). The HFD + Fru and HFD + Glu groups displayed decreased protein levels of SOD-2, HO-1, and catalase (Figure 2D) and higher TBARS values compared with the ND group (Figure 2E).

### 3.5. Effects of HFD and HFD Supplemented with Fructose or Glucose on Hepatic Apoptosis, Inflammation, and Fibrosis

**Figure 3:**
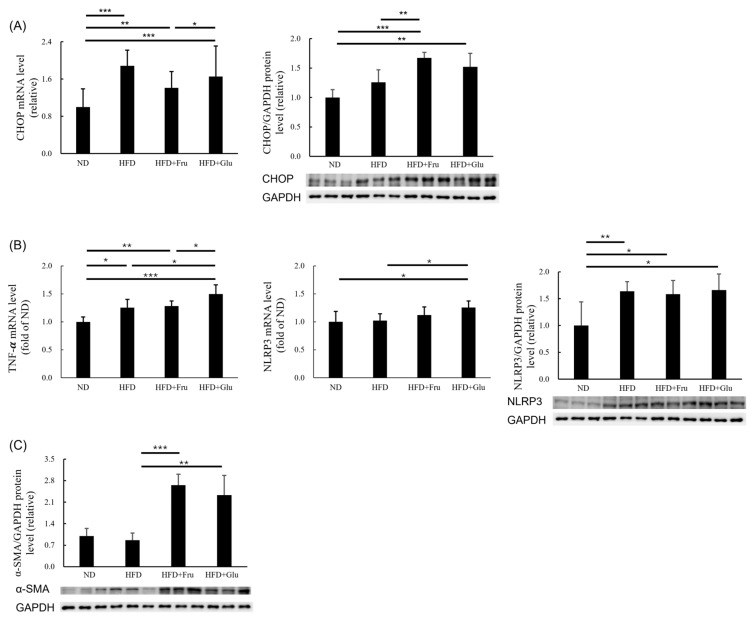
Effects of HFD and HFD supplemented with fructose or glucose on hepatic apoptosis, inflammation, and fibrosis. (**A**) mRNA and protein levels of CHOP. (**B**) mRNA levels of TNF-α and NLRP3 and protein level of NLRP3. (**C**) Protein level of αSMA. ND, normal diet; HFD, high-fat diet; HFD + Fru, high-fat diet supplemented with fructose; HFD + Glu, high-fat diet supplemented with glucose; α-SMA, α-smooth muscle actin; CHOP, C/EBP homologous protein; NLRP3, nucleotide-binding oligomerization domain leucine-rich repeat and pyrin domain containing 3; TNF-α, tumor necrosis factor α. * *p* < 0.05, ** *p* < 0.01, *** *p* < 0.001.

The HFD, HFD + Fru, and HFD + Glu groups displayed increased CHOP mRNA expression compared with the ND group, and the HFD + Fru and HFD + Glu groups displayed increased protein levels of CHOP compared with the HFD and ND groups (Figure 3A). The HFD, HFD + Fru, and HFD + Glu groups displayed increased TNF-α mRNA expression compared with the ND group, with the HFD + Glu group having the highest TNF-α expression. Furthermore, the HFD + Fru and HFD + Glu groups had higher increased protein levels of NLRP3 than did the ND group, and the HFD + Glu group also exhibited a higher increased NLRP3 mRNA expression than did the ND group (Figure 3B). The α-SMA protein level was higher in the HFD + Fru and HFD + Glu groups than in the HFD group, and no difference in the α-SMA protein level was detected between the ND and HFD groups (Figure 3C).

### 3.6. Effects of HFD and HFD Supplemented with Fructose or Glucose on Hepatic Ceramides

**Figure 4:**
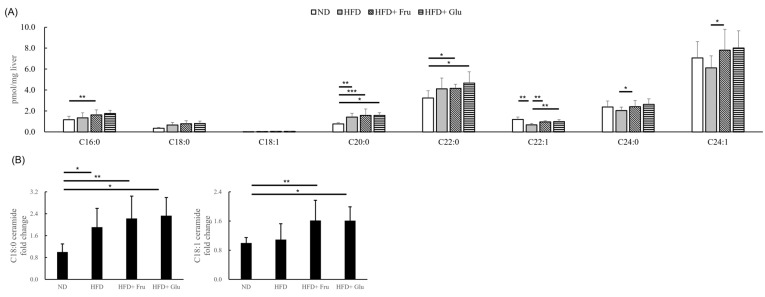
Effects of HFD and HFD supplemented with fructose or glucose on hepatic ceramides. (**A**) Levels of C16:0, C16:1, C18:0, C18:1, C20:0, C22:0, C22:1, C24:0, and C24:1 ceramides. (**B**) Levels of C18:0 and C18:1 ceramides. ND, normal diet; HFD, high-fat diet; HFD + Fru, high-fat diet supplemented with fructose; HFD + Glu, high-fat diet supplemented with glucose. * *p* < 0.05, ** *p* < 0.01, *** *p* < 0.001.

We analyzed nine hepatic ceramide species, namely C16:0, C16:1, C18:0, C18:1, C20:0, C22:0, C22:1, C24:0, and C24:1. Because of its concentration being below the detection limit, C16:1 ceramide is not shown. We observed a general trend of increasing levels of C16:0, C18:0, C18:1, C20:0, and C22:0 ceramides, with the highest levels found in the HFD + Fru and HFD + Glu groups, followed by the HFD group and then the ND group. No differences in ceramide levels were noted between the HFD + Fru and HFD + Glu groups (Figure 4A). Because of the low levels of C18 and C18:1 ceramides, we present their fold changes in Figure 4B.

### 3.7. Result of Clinical Study

**Figure 5:**
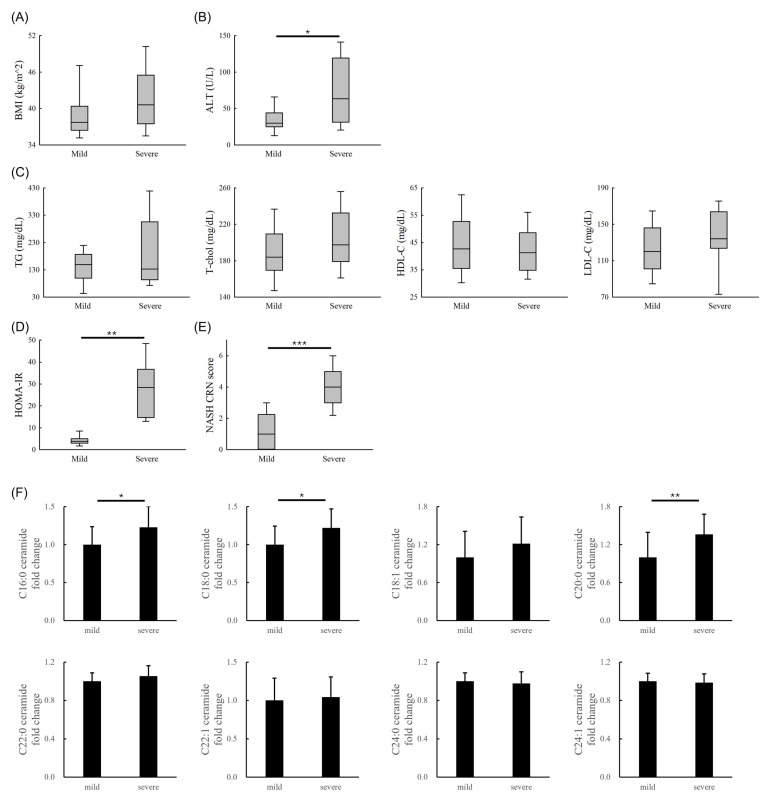
Clinical study results. (**A**) BMI. (**B**) ALT. (**C**) TG, T-chol, HDL-C, and LDL-C levels. (**D**) Insulin resistance. (**E**) NASH CRN score. (**F**) Hepatic ceramide levels. The “mild” group refers to patients with a NASH CRN steatosis score of 0 or 1, whereas the “severe” group includes those with a NASH CRN steatosis score of 2 or 3 or metabolic dysfunction-associated steatohepatitis; ALT, alanine aminotransferase; BMI, body mass index; Cer, ceramide; HDL-C, high-density lipoprotein cholesterol; HOMA-IR, homeostasis model assessment-insulin resistance; LDL-C, low-density lipoprotein cholesterol; NASH CRN, nonalcoholic steatoheaptitis Clinical Research Network; T-chol, total cholesterol; TG, triglyceride. * *p* < 0.05, ** *p* < 0.01, *** *p* < 0.001.

Thirty-nine patients with morbid obesity who were receiving bariatric surgery were included in this study. We categorized the patients into two groups on the basis of steatosis severity: mild (fatty liver with mild steatosis and a NASH CRN steatosis score of 0 or 1, *n* = 18) or severe (fatty liver with marked steatosis and a NASH CRN steatosis score of 2 or 3 or MASH, *n* = 21). The severe group had higher ALT (Figure 5B), greater insulin resistance (Figure 5D), and higher NASH CRN scores (Figure 5E) than did the mild group. In addition, the severe group tended to have higher BMI and higher total cholesterol and LDL-C levels than did the mild group.

We analyzed the same nine hepatic ceramides (C16:0, C16:1, C18:0, C18:1, C20:0, C22:0, C22:1, C24:0, and C24:1) in these patients with obesity. Given the small tissue specimens and low ceramide levels, we present the fold changes in ceramides compared with the mild group. Overall, the ceramide levels revealed little change in these patients. The severe group had higher C16:0, C18:0, and C20:0 ceramide levels than did the mild group (Figure 5F). The results are close to the findings in the animal investigations, where C16:0, C18:0, and C20:0 ceramide levels were higher in the HFD, HFD + Fru, and HFD + Glu groups than in the ND group (Figure 4A).

## 4. Discussion

Compared with the ND, the HFD increased hepatic steatosis, plasma lipid levels, steatosis-related hepatic inflammation, and early-stage liver fibrosis by elevating lipid synthetic enzymes, oxidative stress, apoptosis, and inflammatory cytokines. Supplementation with fructose or glucose exacerbated liver damage, but no significant differences were detected between the impacts of glucose and fructose addendum. The expression patterns of hepatic ceramides in mice nourished on an HFD, whether added with glucose or fructose, were similar to those observed in patients with obesity who had severe hepatic steatosis or MASH. These findings suggest the involvement of ceramides in the progression of hepatic lipotoxicity.

The HFD + Glu group consumed more water than did the other groups (Figure S1B). This result is similar to that of a study in which mice fed glucose water drank more than did mice fed fructose water [20]. Our study compared the effects of fructose and glucose under similar caloric status. Thus, we adjusted the glucose concentration at weeks 2 and 6. After these adjustments, the caloric intake was similar between the HFD + Fru and HFD + Glu groups (Figure S1C).

In hepatic DNL, insulin induces SREBP-1c, which transcriptionally stimulates genes engaged in fatty acid synthesis. ACC and FAS are key enzymes in DNL [31]. Because of substantial steatosis and increased protein levels of SREBP-1c and ACC in the HFD + Fru and HFD + Glu groups, the mRNA expression levels of SREBP-1c and ACC were downregulated. A phase II trial reported that patients who received an FAS inhibitor (TVB-2640) exhibited decreased liver fat content [32]. The observed decrease in FAS mRNA and protein levels in the HFD, HFD + Fru, and HFD + Glu groups, despite repeated measurements, is difficult to explain. In addition, the PPAR-α protein level was downregulated in the HFD + Fru and HFD + Glu groups (Figure 2B); this finding is consistent with that of another study [33]. In this study, we identified that the SOD-2, HO-1, and catalase protein levels were downregulated in the animal model nourished an HFD with or without glucose or fructose supplementation (Figure 2D). Moreover, the TBARS values were higher in the HFD + Fru and HFD + Glu groups (Figure 2E). The protein levels of p-Nrf2, GCLM, and GCLC were increased in the HFD and HFD + Fru groups but decreased in the HFD + Glu group. In addition, the GPX2 protein level was increased in the HFD, HFD + Fru, and HFD + Glu groups. These glutathione precursors may be upregulated as a compensatory response to oxidative stress in mice with hepatic steatosis and inflammation (Figure 2C). Increased hepatic apoptosis, necroinflammation, and fibrosis in the HFD, HFD + Fru, and HFD + Glu groups were indicated by elevated mRNA and protein levels of CHOP, TNF-α, NLRP3, and α-SMA (Figure 3).

Softic et al. demonstrated that mice fed an HFD with added fructose had more obvious obesity, insulin resistance, and hepatomegaly compared with mice fed an HFD with added glucose. In their study, mice could drink water freely (30% *w*/*v* glucose or fructose), and the mice that received an HFD with added glucose consumed approximately twice as much water as did the mice that received an HFD with added fructose [20]. In this study, we noted that additional carbohydrate energy resulted in increased liver damage; however, when comparing isocaloric intake, fructose and glucose caused similar levels of liver damage.

Pancreatic β-cell insulin secretion is not stimulated by fructose [34]. However, fructose contributes to hepatic insulin resistance through increased accumulation of hepatic diacylglycerol, activation of protein kinase C, impairment of insulin-mediated Akt2 activation, and blocking of hepatic β-fatty acid oxidation [35,36]. The findings suggest that fructose works as a pure lipogenic molecule in the liver. Although small amounts of dietary fructose increase hepatic glucose uptake through glucokinase activation [37], overt fructose intake is related to impaired glucose uptake and decreased glucokinase activity and liver glycogen content [38]. However, human interventional studies comparing the impacts of glucose and fructose on insulin resistance and hepatic fat content have not demonstrated substantial differences [13,14,15,39,40,41]. These intervention periods were relatively short (ranging from 7 days to 10 weeks), and the long-lasting diverse impacts of glucose and fructose on MASLD progression remain inconclusive. In summary, the debate regarding the distinct impacts of glucose and fructose on MASLD deterioration continues.

Emerging data indicate that glucagon-like peptide-1 receptor agonists could alleviate liver steatosis and DNL in patients with obesity [42]. Resmetirom, a liver-directed thyroid hormone receptor, revealed promising results in this context [43]. Although pharmacological interventions are being explored, lifestyle modification and dietary changes remain effective alternatives for managing fatty liver disease. Fucho et al. reported that C16 ceramide induces oxidative stress and impairs fatty acid oxidation, resulting in lipid accumulation in the liver [44]. In addition, in individuals with obesity, overexpression of ceramide synthase 6 mRNA in adipose tissue has been correlated with insulin resistance [45]. Ceramides can also promote the generation of reactive oxygen species in cultured hepatocytes by impairing mitochondrial electron transport activities [46]. Our animal study demonstrated a trend of increasing levels of C16:0, C18:0, C18:1, C20:0, and C22:0 ceramides, with the highest levels found in the HFD + Fru and HFD + Glu groups, followed by the HFD group and the ND group. Similarly, patients with obesity with severe fatty liver or MASH had higher C16, C18, and C20 ceramide levels (by an average of 22–36%) than did those with mild fatty liver. The increased ceramide may come from the accumulation of serine and palmitoyl-CoA. The synthesis of ceramide is activated by oxidative stress, inflammation, and overt free fatty acids [47]. The patients also had higher ALT and NASH CRN scores than did those with mild steatosis. However, we cannot ascertain whether patients with severe steatosis had been on a high carbohydrate diet. These findings suggest that ceramides could be an imaginable therapeutic target in the subsequent study.

## 5. Conclusions

The HFD increased hepatic steatosis, plasma lipid levels, steatosis-related hepatic inflammation, and early-stage liver fibrosis. Supplementation with fructose or glucose exacerbated liver damage, but no significant disparity between the influences of fructose and glucose supplementation were noted. Dietary composition, including ceramides, might be a promising therapy in the subsequent study.

## Data Availability

The data presented in this study are available upon reasonable request from the corresponding author.

## Supplementary Materials

The following supporting information can be downloaded at: https://www.mdpi.com/article/10.3390/nu16203571/s1, Figure S1: Food, water, and energy intake of mice; Table S1: Primers used in this study; Table S2: Amount of food and drinking water consumed per mouse in each group; Table S3: NASH CRN scores in each group.
