# Supplementary material for: No Difference in Liver Damage Induced by Isocaloric Fructose or Glucose in Mice with a High-Fat Diet
Source: Nutrients. 2024 Oct 21;16(20):3571. doi: 10.3390/nu16203571 (PMC11510609; doi:10.3390/nu16203571)
Supplement: Supplementary file 1 [file nutrients-16-03571-s001.zip › nutrients-3227209-supplementary.pdf]

Supplementary materials

No difference in liver damage induced by a high-fat diet supplemented with isocaloric fructose or glucose

Wei-Fan Hsu, Ming-Hsien Lee, Chong-Kuei Lii, and Cheng-Yuan Peng

Table S1. Primers used in this study

Gene name	forward	reverse
SREBP-1c	5′-CGTCTGCACGCCCTAGG-3′	5′-CTGGAGCATGTCTTCAAATGTG-3′
ACC	5′-TGACAGACTGATCGCAGAGAAAG-3′	5′-TGGAGAGCCCCACACACA-3′
FAS	5′-GCTGCGGAAACTTCAGGAAAT-3′	5′-AGAGACGTGTCACTCCTGGACTT-3′
C/EBPα	5′- GGGCACTGACCACTCTGTT-3′	5′- CACGCACATCCCAAAGCC -3′
TNF-α	5′-ATGAGAAGAGGCTGAGACA -3′	5′-AGACACCATGAGCACAGA-3′
NLRP3	5′-GAAGAAGAGAGGAGAGGAGGTCG-3′	5′-TTCACCAGTCTGGAAGAACAGGCAAC-3′
GAPDH	5′-GCCTGGAGAAACCTGCCAAGTATG-3′	5′-GGGAGTTGCTGTTGAAGTCGCA-3′

ACC, acetyl-CoA carboxylase; FAS, fatty acid synthase; GAPDH, glyceraldehyde-3-phosphate dehydrogenase; NLRP3, nucleotide-binding oligomerization domain leucine-rich repeat and pyrin domain containing 3; SREBP, sterol regulatory element-binding protein; TNF-α, tumor necrosis factor α

Table S2. Amount of food and drinking water consumed per mouse in each group

Variable (n = 12)	ND	HFD	HFD+Fru	HFD+Glu	*P* [a]
Food (g/day)	2.33± 0.30	2.13± 0.25	1.87± 0.24	1.83± 0.26[a]	< 0.001

	(3.8 kcal/g)	(5.2 kcal/g)	(5.2 kcal/g)	(5.2 kcal/g)	
Drinking water (g/day)	2.64± 0.31	2.41± 0.31	2.47± 0.39	7.49± 1.37	< 0.001
Total calories (kcal/day)	8.84± 1.15	11.06± 1.29***	12.68± 1.45***###	12.94± 1.71***###	< 0.001

ND, normal diet; HFD, high-fat diet with tap water; HFD+Fru, high-fat diet supplemented with fructose water; HFD+Glu, high-fat diet supplemented with glucose water.

[a] Statistical analysis was performed using one-way analysis of variance.

***$p < 0.001$ versus chow group; ###$p < 0.01$ versus HFD group

Table S3. NASH CRN scores in each group

Variable (n = 12)	ND	HFD	HFD+Fru	HFD+Glu	P^a
Steatosis	0.98± 0.47	2.42± 0.79***	2.26± 0.70***	2.75± 0.44***#b	< 0.001
Lobular inflammation	0.22± 0.42	0.23± 0.43	0.70± 0.68***###	0.37± 0.49c	0.001
Hepatocyte ballooning	0.62± 0.49	1.45± 0.72***	1.33± 0.66***	1.55± 1.50***	< 0.001
NAS	1.80± 0.92	4.08± 1.53***	4.30± 1.40***	4.67± 0.75***#	< 0.001

NASH CRN, nonalcoholic steatohepatitis Clinical Research Network; ND, chow diet; HFD, mice fed on a high-fat diet supplemented with tap water; HFD+Fru, mice fed on HFD supplemented with fructose water; HFD+Glu, mice fed on HFD supplemented with glucose water.

[a] one-way ANOVA test

***$p < 0.001$ versus chow group; #$p < 0.05$ versus HFD group; ###$p < 0.001$ versus HFD group; [b]$p < 0.001$ versus HFD+Fru group; [c]$p < 0.01$ versus HFD+Fru group

Supplementary figure legends

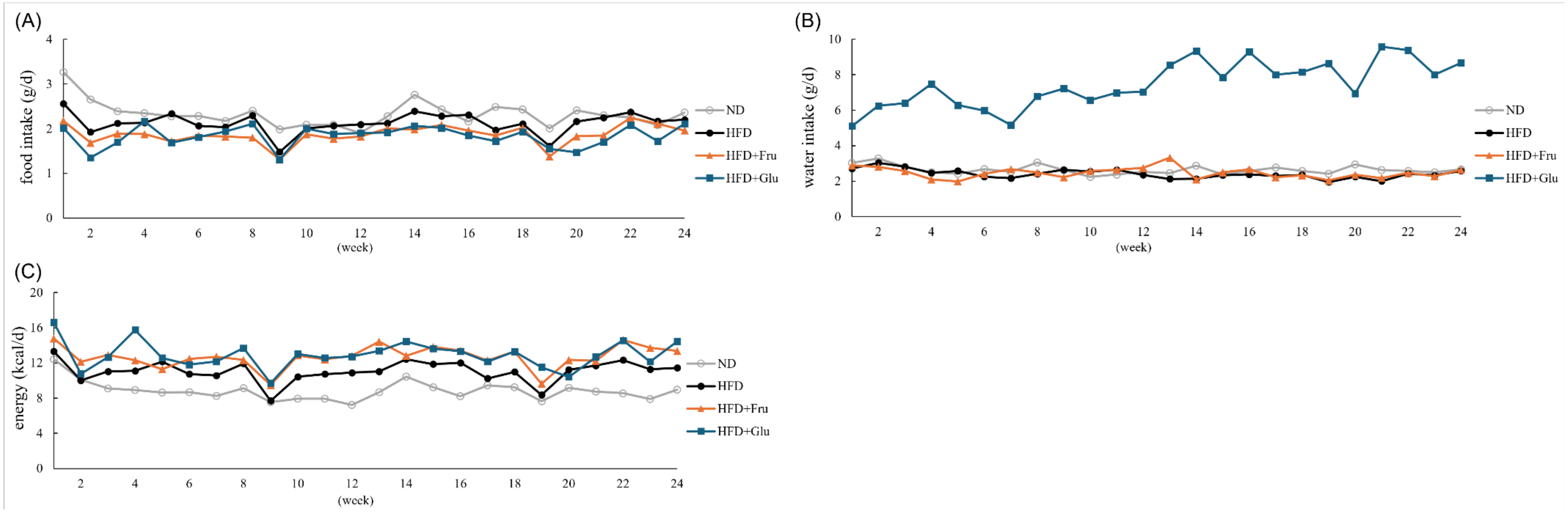

Figure S1. Food, water, and energy intake of mice. (A) Daily food intake (g/d). (B) Daily water intake (g/d). (C) Daily energy intake (kcal/d). ND, normal diet; HFD, high-fat diet; HFD+Fru, high-fat diet supplemented with fructose; HFD+Glu, high-fat diet supplemented with glucose.
